# Effect of laser-induced ultrasound treatment on material structure in laser surface treatment for selective laser melting applications

**DOI:** 10.1038/s41598-021-02895-8

**Published:** 2021-12-06

**Authors:** Ivan A. Ivanov, Vladimir S. Dub, Alexander A. Karabutov, Elena B. Cherepetskaya, Anton S. Bychkov, Igor A. Kudinov, Artem A. Gapeev, Mikhail D. Krivilyov, Nikolay N. Simakov, Svetlana A. Gruzd, Stepan L. Lomaev, Vladimir V. Dremov, Pavel V. Chirkov, Roman M. Kichigin, Alexey V. Karavaev, Maxim Yu. Anufriev, Konstantin E. Kuper

**Affiliations:** 1 JSC “NPO “TSNIITMASH”, 4 Sharikopodshipnikovskaya St., Moscow, 115088 Russia; 2 LLC “Rusatom-Additive Technology” Industrial integrator the SC Rosatom, 49 Kashirskoye highway, Moscow, 115409 Russia; 3 The National University of Science and Technology MISiS, 4 Leninskiy Prospect, Moscow, 119991 Russia; 4 Lomonosov Moscow State University, 1 Leninskie Gory, Moscow, 119991 Russia; 5 The Institute on Laser and Information Technologies-Branch of the FSRC Crystallography and Photonics of Russian Academy of Sciences, 1 Svyatoozerskaya St., Shatura, Moscow, 140700 Russia; 6 Udmurt State University, Universitetskaya str. 1, Izhevsk, 426034 Russia; 7 Udmurt Federal Research Center of the Ural Branch of RAS, Baramzina str. 34, Izhevsk, 426067 Russia; 8 Federal State Unitary Enterprise “Russian Federal Nuclear Center-Zababakhin All-Russia Research Institute of Technical Physics”, 13 Vasiliev st., Snezhinsk, 456770 Russia; 9 Snezhinsk Physics and Technology Institute, National Research Nuclear University “MEPhI” (Moscow Engineering Physics Institute), Snezhinsk, 454070 Russia; 10 Budker Institute of Nuclear Physics, Siberian Branch Russian Academy of Sciences, Novosibirsk, 630090 Russia

**Keywords:** Atomistic models, Coarse-grained models, Structure of solids and liquids, Design, synthesis and processing, Solid-state lasers, Laser material processing, Photoacoustics

## Abstract

A new mechanism for controlling the microstructure of products in manufacturing processes based on selective laser melting is proposed. The mechanism relies on generation of high-intensity ultrasonic waves in the melt pool by complex intensity-modulated laser irradiation. The experimental study and numerical modeling suggest that this control mechanism is technically feasible and can be effectively integrated into the design of modern selective laser melting machines.

## Introduction

Additive manufacturing (AM) of parts with complex shapes has been developing intensively in recent decades. However, despite the variety of AM process, including selective laser melting (SLM)^1–3^, direct laser metal deposition^4–6^, electron beam melting^7,8^ and others^9,10^, the manufactured parts may contain imperfections. This is primarily due to the specific features of the melt pool solidification process associated with high thermal gradients, high cooling rates, and the complexity of heating cycles in the melted and re-melted material^11^, which lead to epitaxial grain growth and significant porosity^12,13^. It was shown^13^ that it is necessary to control either the thermal gradients, cooling rates, and alloy composition, or to apply additional physical impact by external fields of various nature (e.g. ultrasonic), in order to achieve fine equiaxed grain structure.

A significant number of publications are related to the effects of vibration treatment on the solidification process during conventional casting^14,15^. However, exerting external fields on large volumes of melt do not result in desired microstructure of the material. The situation changes drastically if the volume of the liquid phase is small. In this case, external fields significantly affect the solidification process. Intense acoustic fields^16–27^, arc stirring^28^ and oscillations^29^, electromagnetic effects during a pulsed plasma arc^30,31^ and other methods^32^ have been considered. Grain refinement of the microstructure by several times was observed during laser-based direct energy deposition of Ti-6Al-4V^26,33^ alloy, nickel-based superalloy Inconel 625^26^ and 316L stainless steel^34^ with an external high-intensity ultrasound source (a sonotrode vibrated at 20 kHz) attached to the substrate. The ultrasound-induced grain refinement is attributed to the increased constitutional supercooling zone due to the lowered temperature gradient and ultrasound-enhanced generation of new crystallites through cavitation^34^.

In this work, we investigate the possibility of changing the grain structure of austenitic stainless steel by ultrasonic treatment of the melt pool with acoustic waves generated by the melting laser itself. Intensity modulation of laser radiation incident on an optically absorbing medium can lead to generation of ultrasonic waves that may alter the microstructure of the material. Such an intensity modulation of laser radiation could be easily integrated into the existing SLM 3D printing machines. The experiments in this work were conducted on stainless steel plates, the surface of which was exposed to intensity-modulated laser radiation. Thus, technically, laser surface treatment was performed. However, if such a laser treatment is carried out on the surface of each layer, then during layer-by-layer construction, the effect on the entire volume or on a selected part of the volume is realized. In other words, laser surface treatment of each layer is equivalent to “laser volume treatment” if the part is constructed layer by layer.

Whereas in sonotrode-based ultrasonic treatment, the ultrasonic energy of the standing acoustic wave is distributed throughout the entire part, the intensity of the laser-induced ultrasound is highly localized near the point where laser radiation is absorbed. The use of sonotrode in the SLM powder bed fusion machines is complicated since the top surface of the powder bed, which is exposed to laser radiation, should remain at rest. Moreover, the top surface of the part is mechanically stress-free. Therefore, acoustic stresses are close to zero, and particle velocities have a maximum amplitude over the entire top surface of the part. The acoustic pressures inside the entire melt pool could not exceed 0.1% of the maximum pressure generated by the sonotrode, since the wavelength of ultrasonic waves with frequency of 20 kHz is $\sim 0.3~\text {m}$ in stainless steel, while the depth of the melt pool is typically less than $\sim 0.3~\text {mm}$. Consequently, the effect of the ultrasound on cavitation might be small.

It should be noted, that the use of an intensity-modulated laser radiation in direct laser metal deposition is an active area of research^35–38^.

## Laser ultrasonics and the underlying principles

The thermal effect of laser radiation incident on a medium underlies almost all laser technologies for material processing^39,40^, such as cutting^41^, welding, hardening, drilling^42^, surface cleaning, surface alloying, surface polishing^43^ etc. The invention of lasers stimulated the development of new technologies for material processing, and the first results were summarized in a number of reviews and monographs^44–46^.

It should be noted that any non-stationary action on a medium, including laser action on an absorbing medium, leads to the excitation of acoustic waves in it with greater or lesser efficiency. At first, the main attention was paid to laser excitation of waves in a liquid and various mechanisms of thermal excitation of sound (thermal expansion, evaporation, volume change during a phase transition, striction, etc.)^47–49^. A theoretical analysis of this process and its possible practical applications have been presented in a number of monographs^50–52^.

Subsequently, these problems were discussed at various conferences, and laser excitation of ultrasound found its application both in industrial applications of laser technologies^53^ and in medicine^54^. Therefore, the basic concepts of the processes occurring under pulsed laser action on absorbing media can be considered established. Laser ultrasonic inspection was used for defect detection in SLM manufactred samples^55,56^.

The impact on the material of a laser-generated shock wave is the basis of laser shock peening^57–59^, which has also been used for surface treatment of additively manufactured parts^60^. However, laser shock peening is most effective with nanosecond laser pulses and a mechanically loaded surface (for example, with a layer of liquid)^59^, since the mechanical loading increases the peak pressure.

## Experimental setup

**Figure 1:**
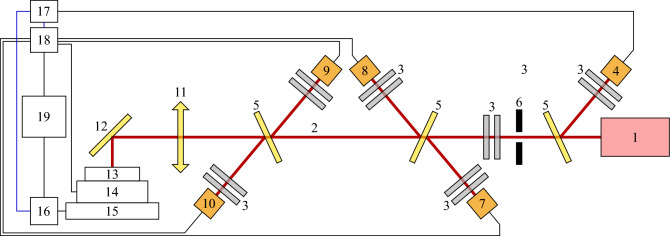
Functional schematic of the experimental setup: 1—laser; 2—laser beam; 3—neutral density filters; 4—synchronization photodiode; 5—beam splitting plates; 6—diaphragm; 7—calorimeter of the incident beam; 8—calorimeter of the reflected beam; 9—power meter of the incident beam; 10—power meter of the reflected beam; 11—focusing lens; 12—mirror; 13—sample; 14—wideband piezoelectric transducer; 15—2D translator; 16—positioning microcontroller; 17—synchronization unit; 18—multi-channel digital acquisition system with various sampling rates; 19—personal computer.

An experiment was carried out to study the possible effect of various physical fields on the microstructure of the solidified material. The functional diagram of the experimental setup is shown in Fig. 1. A pulsed Nd:YAG solid-state laser operating in a free-running mode (pulse duration $\tau _L \sim 150~\upmu \text {s}$) was used. Each laser pulse passed through a series of neutral density filters and a system of beam-splitting plates. The pulse energies on a target varied from $E_L \sim 20~\text {mJ}$ up to $E_L \sim 100~\text {mJ}$ depending on the combination of neutral density filters. The laser beams reflected from the beam-splitting plates were fed to the photodiode used to syncronize the data acquisition, two calorimeters (photodiodes with long response times exceeding $1~\text {ms}$) for determining the optical energy incident on the target and reflected from it, and two power meters (photodiodes with short response times $<10~\text {ns}$) for determining the incident and reflected optical power. The calorimeters and power meters were calibrated using a termopile detector Gentec-EO XLP12-3S-H2-D0 and a dielectric mirror mounted in place of the sample to give the values in absolute units. The beam was focused onto the target using a lens (antireflection coating at $1.06 \upmu \text {m}$, focal length $160~\text {mm}$), and the beam waist at the target surface was 60–$100~\upmu \text {m}$.

**Figure 2:**
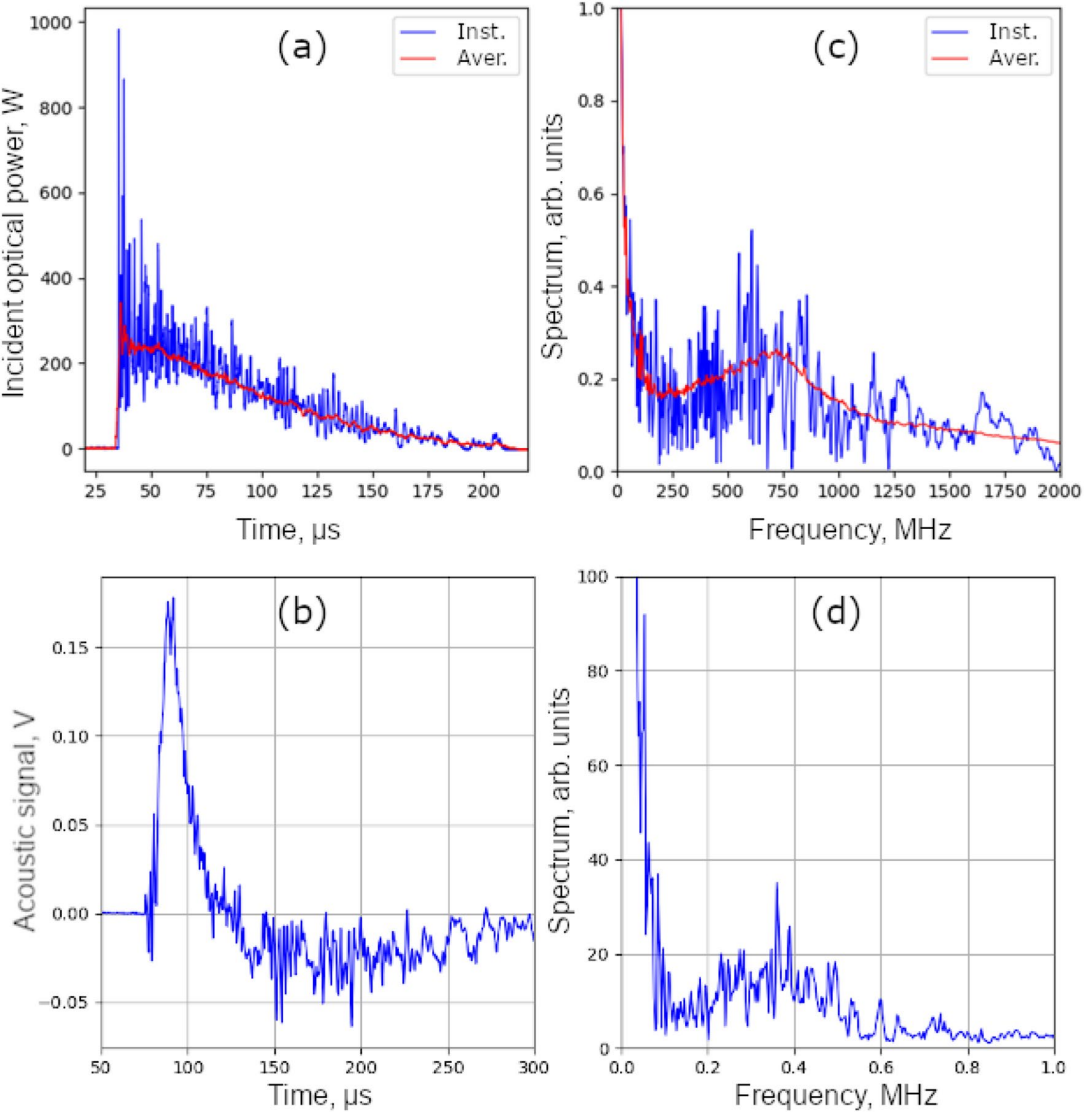
Temporal profiles of the laser pulse intensity (**a**) and the acoustic velocity at the rear surface of the sample (**b**), the spectra of the laser pulse (**c**) and the ultrasonic pulse (**d**) for a single laser pulse (blue curves) and averaged over 300 laser pulses (red curves).

Ultrasonic treatment was done as follows. The laser operated in the free-running mode; so the laser pulse had a duration of $\tau _L \sim 150~\upmu \text {s}$ and consisted of a multitude of shorter pulses with a duration of about $1.5~\upmu \text {s}$ each. The temporal shape of the laser pulse and its spectrum, consisting of a low-frequency envelope and high-frequency modulation with an average frequency of about $0.7~\text {MHz}$, are shown in Fig. 2. The low-frequency envelope provided heating with subsequent melting and evaporation of the material, while the high-frequency component provided the generation of ultrasonic vibrations due to photoacoustic effect. The waveform of the laser-generated ultrasonic pulse is mainly determined by the temporal shape of the laser pulse intensity, which provides a broadband ultrasonic treatment of the sample in the frequency range from $7~\text {kHz}$ up to $2~\text {MHz}$ with a central frequency of $~0.7~\text {MHz}$. The acoustic pulse generated due to the photoacoustic effect was recorded using a broadband piezoelectric transducer made of a polyvinylidene fluoride film. The recorded waveform and its spectrum are shown in Fig. 2. It should be noted, the waveform of the laser pulse is typical for lasers in the free-running mode^61^.

We can clearly distinguish the low-frequency and high-frequency components of acoustic treatment corresponding to the low-frequency envelope and high-frequency modulation of the laser pulse, respectively. The wavelength of the acoustic waves generated by the laser pulse envelope exceeded $40~\text {cm}$; therefore, the main effect on the microstructure was expected from the broadband high-frequency component of the acoustic signal.

## Macroscopic simulation of thermal and deformation loads

Physical processes in SLM are complex and occur simultaneously at different spatial and time scales. Hence, multiscale approach is mostly suitable for theoretical analysis of SLM. The mathematical models should be initially multi-physical. Then the mechanics and thermophysics of a multiphase medium “solid phase–liquid melt” which interacts with inert gas atmosphere can be efficiently described^62^. The features of thermal load of material in SLM are as follows.

1. High heating and cooling rates up to $10^6~\text {K}/\text {s}$ owing to localized laser irradiation with the power density up to $10^{13}~\text {W}/\text {cm}^2$.
2. A size of the heat-affected zone correlates with the efficient beam diameter.
3. Due to warming-up of the substrate, a properly selected delay between separate scans is required.
4. The melting-solidification cycle lasts between 1 and $10~\text {ms}$ which facilitates rapid solidification of the molten zone during cooling.

Rapid heating of the sample’s face results in formation of high thermo-elastic stresses in the surface layer. Sufficient (up to 20%) fraction of the powder layer is intensively evaporated^63^ that leads to additional pressure load on the surface in response to laser ablation. Consequently, the induced strains significantly distort the part’s geometry, especially in the vicinity of supports and thin structural elements. The high heating rate in pulse laser annealing leads to generation of an ultrasonic strain wave which propagates from the surface to the substrate. To get accurate quantitative data on local stress and strain distributions, mesoscopic simulation of a problem of elastic deformation conjugated with heat and mass transfer was performed.

The governing equations of the model include (1) unsteady heat transfer equation with the thermal conductivity dependent on the phase state (powder, melt, polycrystal) and temperature, (2) wave equation for elastic deformation of continuous medium after ablation and thermoelastic expansion. The boundary-value problem was determined from experimental conditions. The modulated laser flux was defined on the sample surface. Convective cooling includes both conductive thermal exchange and evaporation flux. The mass flux was defined based on calculation of the saturated vapor pressure of evaporated material. The elasto-plastic stress–strain relation was used where the thermoelastic stress is proportional to the temperature difference. The simulations were carried out for parameters of a pulsed laser with the nominal power of $300~\text {W}$, frequency of $10^5~\text {Hz}$, intermittency factor of 100, and effective beam diameter of $200~\upmu \text {m}$.

**Figure 3:**
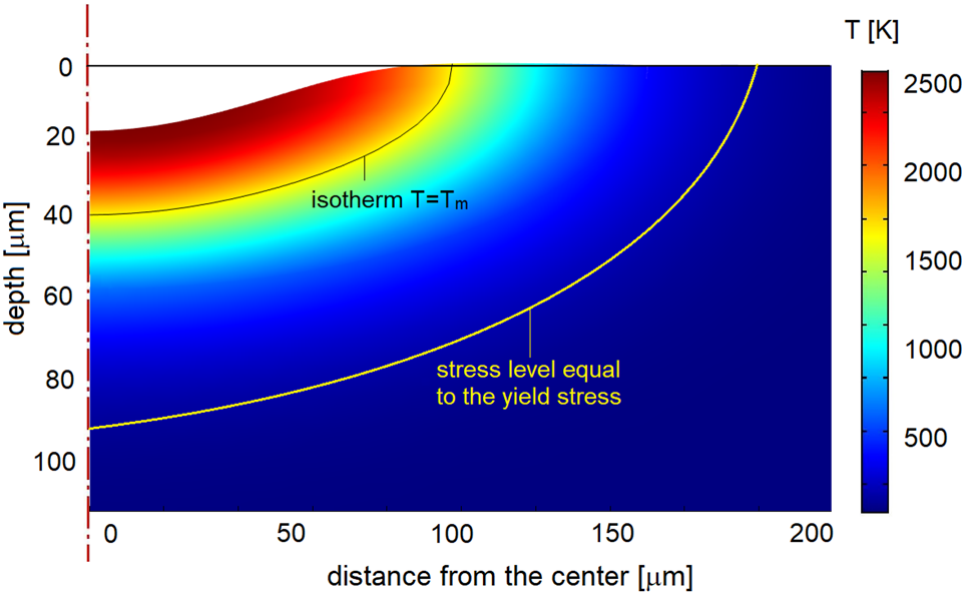
The results of numerical simulation of the molten zone for a single laser pulse annealed on a 316L sample plate. The time is $180~\upmu \text {s}$ from the pulse start when the depth of the molten pool reaches its maximum. The isotherm $T = T_L = 1723~\text {K}$ represents the boundary between the liquid and solid phases. The isobar (yellow line) corresponds to the yield stress calculated as a function of temperature in the next section. Thus, in the domain between two isolevels (isotherm $T=T_L$ and isobar $\sigma =\sigma _V(T)$), the solid phase undergoes intensive mechanical load which may result in microstructure modification.

The results of numerical simulation of the molten zone using the macroscopic mathematical model are given in Fig. 3. The molten zone is $200~\upmu \text {m}$ in diameter ($100~\upmu \text {m}$ in radius) and $40~\upmu \text {m}$ in depth. The results of simulations reveal that the surface temperature varies in time with the amplitude of $100~\text {K}$ locally thanks to the high intermittency factor of pulse modulation. The heating $V_h$ and cooling $V_c$ rates are of the order of $10^7$ and $10^6~\text {K}/\text {s}$ correspondingly. These values are in close agreement with our previous analysis^64^. The difference in one order of magnitude between $V_h$ and $V_c$ results in fast overheating of the surface layer where thermal conduction to the substrate is not sufficient for heat removal. As a result, at $t=26~\upmu \text {s}$ the surface temperature peaks as high as $4800~\text {K}$. Intensive evaporation of material leads to excessive pressure on the sample’s surface and its denudation.

**Figure 4:**
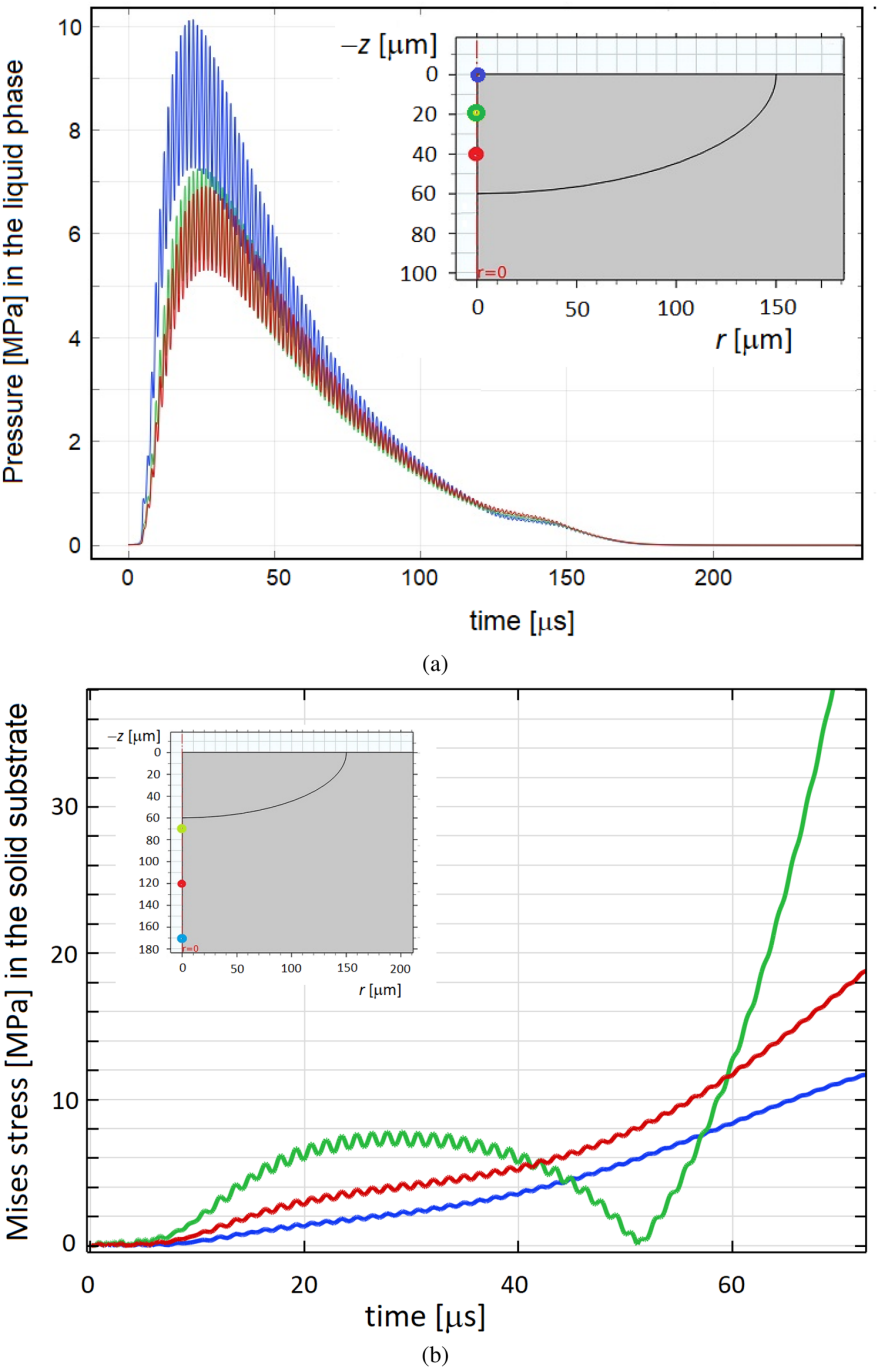
(**a**) Pressure as a function of time calculated along the axis of symmetry at different distances of 0, 20 and $40~\upmu \text {m}$ from the surface. (**b**) Von Mises stress as a function of time calculated in the solid substrate at the distances of 70, 120 and $170~\upmu \text {m}$ from the sample’s surface.

This effect is further explained in Fig. 4a where the pressure level in the molten zone is plotted as a function of time and distance from the surface. First, the pressure behavior correlates with the modulation of intensity of the laser pulse depicted above in Fig. 2. The maximum pressure of about $10~\text {MPa}$ is observed at about $t=26~\upmu \text {s}$. Second, fluctuations of the local pressure in control points have the same oscillatory character with the frequency of $500~\text {kHz}$. It means that the ultrasound pressure wave is generated at the surface and then it travels into the substrate.

The characteristics of the calculated deformation zone in the vicinity of the melted zone are depicted in Fig. 4b. Laser ablation and thermoelastic stresses generate the elastic deformation wave which propagates into the substrate. As follows from the plot, stress generation has two stages. At the first stage at $t < 40~\upmu \text {s}$, the Mises stress rises up to $8~\text {MPa}$ and its modulation is similar to the surface pressure. This stress occurs due to laser ablation and no thermoelastic stresses are observed in the control point since the thermal affected zone is too small initially. When heat dissipates into the substrate, high thermoelastic stress up above $40~\text {MPa}$ develops in the control points.

The obtained *modulated* stress level has a major impact on the solid-liquid interface and can potentially be a governing mechanism in control of the solidification pathway. The deformation zone excesses the melted zone by a factor between 2 and 3 in size. It was shown in Fig. 3 where positions of the melting isotherm and stress level equal to the yield stress are compared. It means that pulse laser irradiation provides high mechanical load in the localized zone with the effective diameter of between 300 and $800~\upmu \text {m}$ depending on the instantaneous time.

Therefore, complex modulation of pulse laser annealing leads to the ultrasonic effect. The microstructure selection pathway is different if compared with SLM without ultrasonic loading. The unsteady zone of deformation leads in periodic cycles of compressions and tensions in the solid phase. Thus, formation of new grain and sub-grain boundaries becomes feasible. Hence, the microstructure characteristics can be intentionally altered as it will be shown below. The obtained conclusion opens possibilities for design of a SLM prototype machine where pulse modulation induces ultrasound actuation. In this case, piezoelectric inductor used elsewhere^26^ can be excluded.

## Results and discussion

### Experimental results

**Figure 5:**
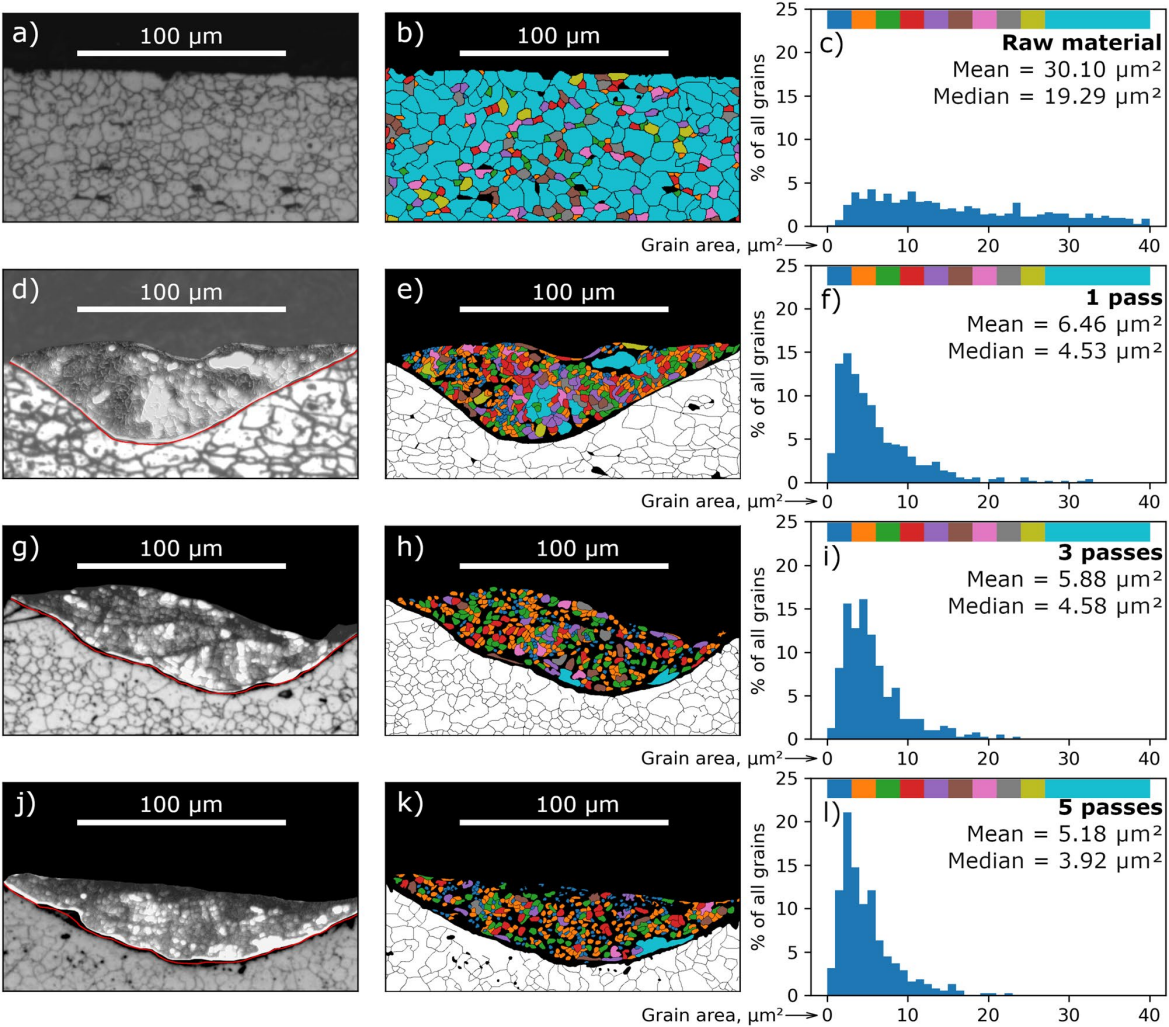
Subfigures (**a**,**d**,**g**,**j**) and (**b**,**e**,**h**,**k**)—the microstructure of the zone subjected to laser melting, subfigures (**c**,**f**,**i**,**l**)—the distribution of the colored grains by area. The coloring indicates the grains that were used to calculate the histograms. The colors correspond to the grain area (see the colorbars at the top of the histograms. Subfigures (**a**–**c**) correspond to untreated stainless steel, subfigures (**d**–**f**), (**g**–**i**), (**j**–**l**) correspond to one, three and five times remelting of the sample, respectively.

The experiments were carried out on AISI 321H stainless steel plates with dimensions of $20 \times 20 \times 5~\text {mm}$. The plate was moved $50~\upmu \text {m}$ after each laser pulse, the laser beam waist at the surface of the target was about $100~\upmu \text {m}$. Up to five subsequent beam passes were performed along the same track to induce remelting of the treated material for the purpose of grain refinement. In all the cases, there was an ultrasonic treatment of the remelting zone, determined by the oscillating component of the laser radiation. This led to a decrease in the average grain area by more than 5 times. Figure 5 shows how the microstructure of the laser-melted area changes with the number of subsequent remelting cycles (passes).

Since the laser pulse energy did not change between subsequent passes, the depth of melted zone was the same. Thus, the subsequent pass completely “overwrote” the previous one. However, the histogram shows that the mean and median value of the grain area decreases with increasing number of passes. That might suggest that the laser is acting on the substrate not the melt.

**Figure 6:**
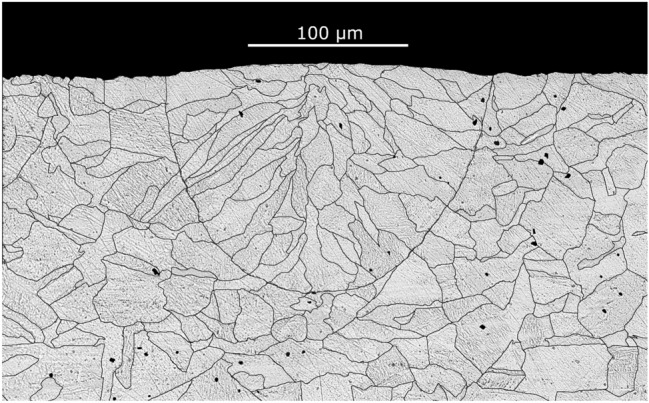
The microstructureof the zone subjected to laser melting by a continuous wave laser (300 W constant power, 200 mm/s scanning speed, AISI 321H stainless steel).

**Figure 7:**
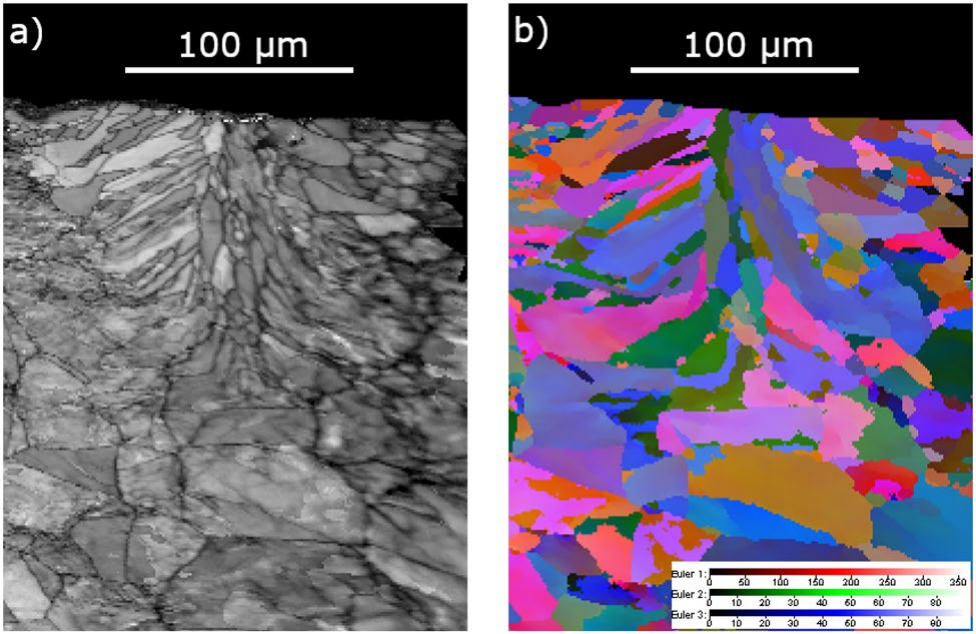
(**a**) The microstructure, and (**b**) the electron back-scatter diffraction image of the zone subjected to laser melting by a continuous wave laser (100 W constant power, 200 mm/s scanning speed, AISI 316L stainless steel) in vacuum $\sim 2~\text {mbar}$.

Grain refinement could be caused by the rapid cooling of the melt pool^65^. Another set of experiments was carried out in which the surfaces of stainless steel plates (321H and 316L) were exposed to continuous-wave laser radiation in atmospheric air (Fig. 6) and in vacuum (Fig. 7). The average laser power (300 W and 100 W, respectively) and the depth of the melt pool were close to those in the experiments with a Nd:YAG laser in a free-running mode. However, a typical columnar structure was observed.

### Microscopic simulation of the yield stress behavior vs temperature

So, it was clearly shown that complex modulation of the laser pulse intensity has a significant effect on the resulting microstructure. We argue that the effect is of mechanical nature and occurs due to the generation of ultrasound vibrations propagating from the irradiated surface of the melt into the depth of the sample. In^13,26,34,66,67^ the similar results were obtained using an external piezoelectric transducer and a sonotrode providing high-intensity ultrasound in various materials including Ti-6Al-4V alloys^26^ and stainless steel^34^. It was supposed that the possible mechanism is as follows. As it was proved in ultrafast in situ synchrotron X-ray imaging intense ultrasound may cause acoustic cavitation^68^. Collapse of the cavitation bubbles in turn produces in the melted material shock waves with pressure at the front reaching about $100~\text {MPa}$^69^. The intensity of such shock waves might be sufficient to facilitate formation of critical size nuclei of the solid phase in the bulk of liquid and thus destruct typical for layer-by-layer additive manufacturing columnar grain structure.

**Figure 8:**
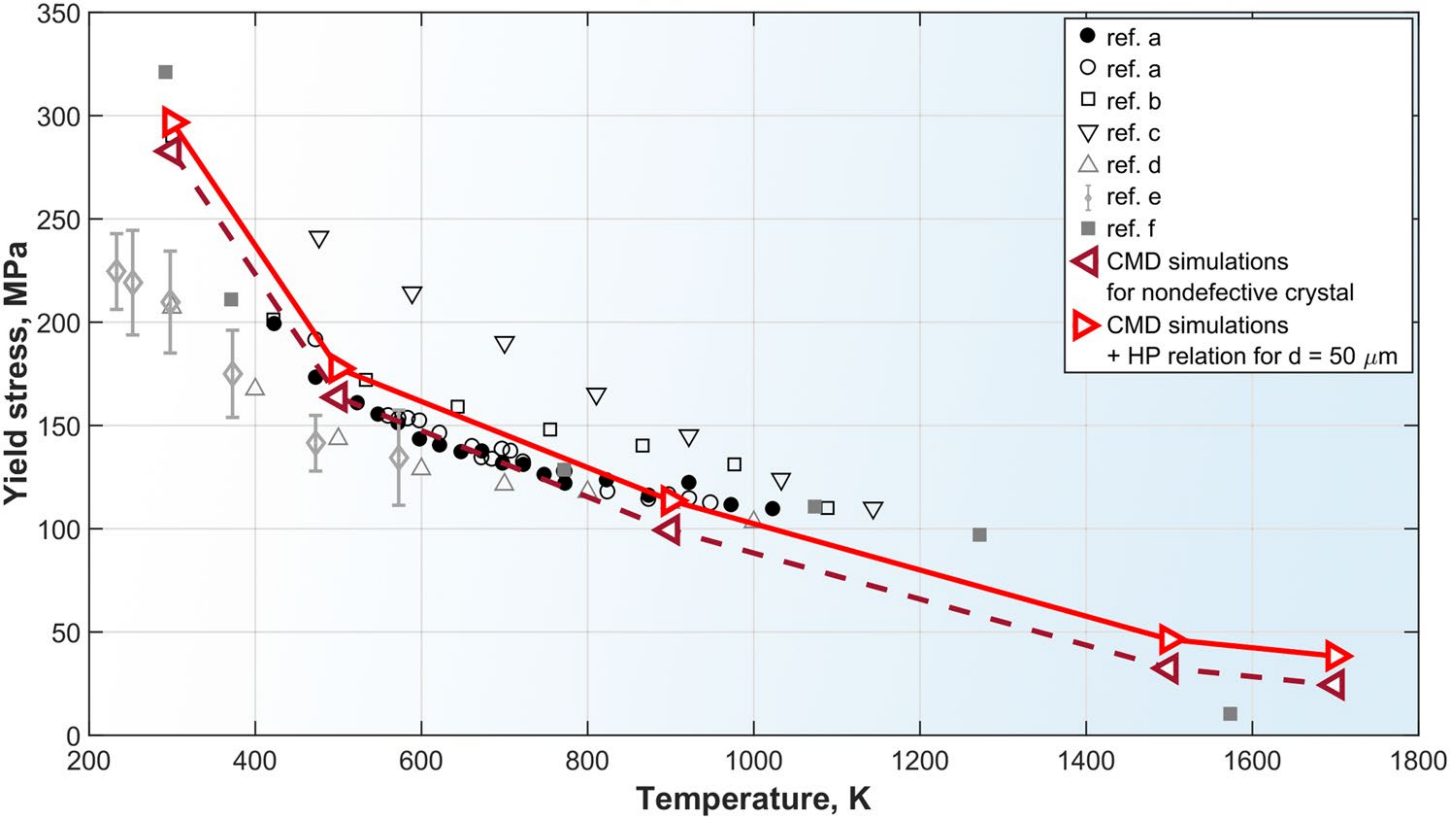
Yield stress of AISI 316 grade austenitic stainless steels and model composition used in MD simulations as function of temperature. Experimental measurements are from references: (**a**)^77^, (**b**)^78^, (**c**)^79^, (**d**)^80^, (**e**)^81^. Ref. (**f**)^82^ is an empirical model for yield stress *vs* temperature dependence used for online stress measurements during laser-aided additive manufacturing. The results of the large-scale MD simulations in the present study are presented as $\vartriangleleft$ for nondefective infinite single crystal and as $\vartriangleright$ taking into account finite grain size via Hall–Petch relation^83^ for an average grain size $d = 50~\upmu \text {m}$.

Here we propose another mechanism responsible for the structure modification by intense ultrasound. The material just after the solidification is at high temperature close to the melting point and has extremely low yield stress. The intense ultrasound may cause plastic flows modifying grain structure of the hot just solidified material. However reliable experimental data on yield stress temperature dependence are available at $T\lesssim 1150~\text {K}$ (see Fig. 8). So, to check the hypothesis we carried out molecular dynamics (MD) simulations of the Fe–Cr–Ni composition similar to steel AISI 316 L with the purpose to evaluate the yield stress behavior in the immediate vicinity of the melting point. To calculate the yield stress we used MD shear stress relaxation technique described in details in^70–73^. For interatomic interaction calculations we used Embedded Atom Model (EAM) from^74^. MD simulations were carried out with LAMMPS code^75,76^. Details of the MD simulations are to be published elsewhere. The results of the MD calculations of the yield stress as a function of temperature are presented in Fig. 8 together with available experimental data and another evaluations^77–82^.

One can see that at $T>1500~\text {K}$ the yield stress dropped below $40~\text {MPa}$. On the other hand, estimations predict the laser generated ultrasound amplitude exceeding $40~\text {MPa}$ (see Fig. 4b) that is quite sufficient to induce plastic flow in the hot just solidified material.

## Conclusion

1. An experimental study of the microstructure formation during SLM of the 12Cr18Ni10Ti (AISI 321H) austenitic stailess steel using a complex intensity-modulated pulsed laser source was carried out.
2. A reduction in grain size in the laser-melted zone was found as a result of sequential laser re-melting after 1, 3 or 5 passes.
3. Macroscopic modeling showed that the estimated size of the zone in which an active effect on the solidification front by ultrasonic deformation is possible is up to $1~\text {mm}$.
4. Microscopic MD modeling showed a significant decrease in the yield strength of the AISI 316 austenitic stainless steel near the melting point down to $40~\text {MPa}$.

The obtained results suggest a method for controlling the material microstructure using complex-modulated laser processing, which can serve as the basis for creating a new modification of the pulsed SLM technology.
